# Supplementary figures and images for: Gelatinase B/matrix metalloproteinase-9 is a phase-specific effector molecule, independent from Fas, in experimental autoimmune encephalomyelitis
Source: PLoS One. 2018 Oct 1;13(10):e0197944. doi: 10.1371/journal.pone.0197944 (PMC6166937; doi:10.1371/journal.pone.0197944)

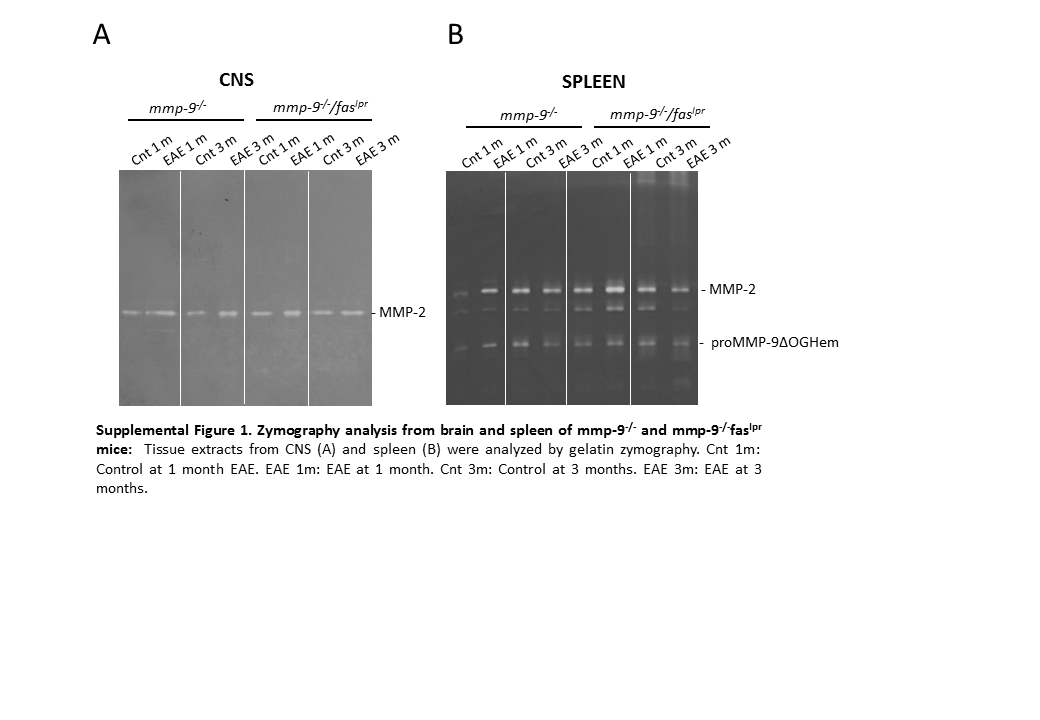

Supplement: S1 Fig — (TIF) [file pone.0197944.s001.tif]

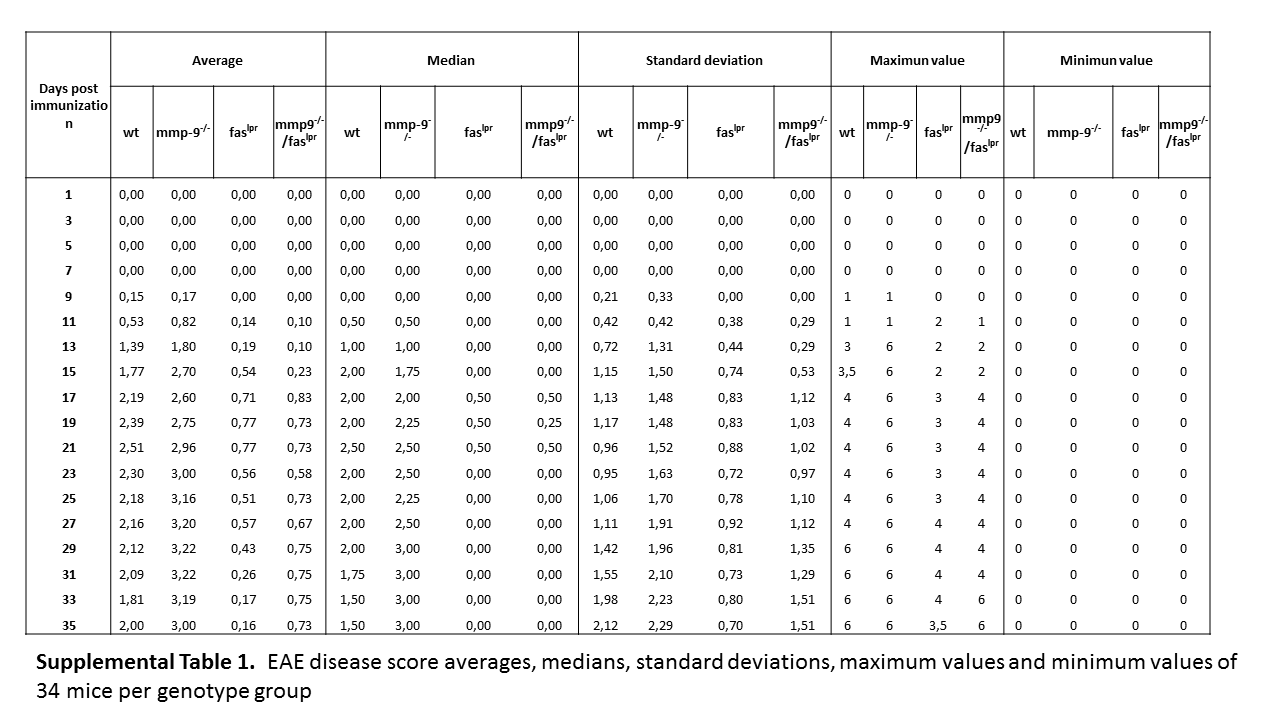

Supplement: S1 Table — (TIF) [file pone.0197944.s002.tif]
